# Detecting Patient Deterioration Early Using Continuous Heart rate and Respiratory rate Measurements in Hospitalized COVID-19 Patients

**DOI:** 10.1007/s10916-022-01898-w

**Published:** 2023-01-24

**Authors:** Guido M Peters, Roel V Peelen, Vincent JHS Gilissen, Mark V Koning, Wim H van Harten, Carine J.M. Doggen

**Affiliations:** 1 Clinical Research Center, Rijnstate Hospital, Arnhem, The Netherlands; 2 Department of Health Technology and Services Research, Technical Medical Centre, University of Twente, Enschede, The Netherlands; 3 Department of Anaesthesiology, Critical Care and Pain Management, Rijnstate Hospital, Arnhem, The Netherlands; 4 Division of Psychosocial Research and Epidemiology, The Netherlands Cancer Institute, Amsterdam, The Netherlands; 5 Rijnstate Hospital, Arnhem, The Netherlands; 6 Scientific Bureau, Rijnstate Hospital, Wagnerlaan 55, PO Box 9555, 6800 TA Arnhem, The Netherlands

**Keywords:** Continuous Monitoring, COVID-19, Deterioration Detection, Wireless Sensor, Heart Rate, Respiratory Rate

## Abstract

**Background:**

Presenting symptoms of COVID-19 patients are unusual compared with many other illnesses. Blood pressure, heart rate, and respiratory rate may stay within acceptable ranges as the disease progresses. Consequently, intermittent monitoring does not detect deterioration as it is happening. We investigated whether continuously monitoring heart rate and respiratory rate enables earlier detection of deterioration compared with intermittent monitoring, or introduces any risks.

**Methods:**

When available, patients admitted to a COVID-19 ward received a wireless wearable sensor which continuously measured heart rate and respiratory rate. Two intensive care unit (ICU) physicians independently assessed sensor data, indicating when an intervention might be necessary (alarms). A third ICU physician independently extracted clinical events from the electronic medical record (EMR events). The primary outcome was the number of true alarms. Secondary outcomes included the time difference between true alarms and EMR events, interrater agreement for the alarms, and severity of EMR events that were not detected.

**Results:**

In clinical practice, 48 (EMR) events occurred. None of the 4 ICU admissions were detected with the sensor. Of the 62 sensor events, 13 were true alarms (also EMR events). Of these, two were related to rapid response team calls. The true alarms were detected 39 min (SD = 113) before EMR events, on average. Interrater agreement was 10%. Severity of the 38 non-detected events was similar to the severity of 10 detected events.

**Conclusion:**

Continuously monitoring heart rate and respiratory rate does not reliably detect deterioration in COVID-19 patients when assessed by ICU physicians.

**Supplementary Information:**

The online version contains supplementary material available at 10.1007/s10916-022-01898-w.

## Introduction

Several studies demonstrated that vital signs such as heart rate, respiratory rate, oxygen saturation, as part of an Early Warning Score (EWS), predict mortality and ICU-admission in COVID-19-patients upon hospital admission [1–6]. Approaches using machine learning techniques are also being developed, which often find heart rate and respiratory rate to be important predictors of deterioration [7–9]. Still, these vital signs are often measured intermittently, leaving a period of time in which deterioration is undetected, creating an opportunity for continuous monitoring to improve detection of patient deterioration [10–12]. In addition, monitoring can be performed remotely, which may reduce nurses’ workload and use of personal protective equipment (PPE). Altogether, continuously monitoring vital signs remotely in patients with COVID-19 may lead to earlier detection of deterioration, reduction of nurses’ workload and a reduced use of PPE.

A variety of commercially available monitoring devices exists. The performance of these devices differs in terms of the vital signs measured, the method of measurement, validity and reliability, as well as signal-handling. However, the utility of a continuous monitoring device may also depend on the underlying disease. In sepsis, for example, an increase in heart rate and respiratory rate is expected, while in COVID-19 hypoxemia is often the only presenting symptom, leaving heart rate and respiratory rate unaffected [13, 14]. Even though EWSs including heart rate and respiratory rate follow disease severity in COVID-19, it is unknown if continuously monitoring these vital signs leads to earlier detection of deterioration, or if it leads to false re-assurance or only false-alarms, leading to alarm fatigue [15–17].

During the pandemic, in our hospital, continuously measuring heart rate and respiratory rate of patients with COVID-19 was mainly used to reduce nurses’ workload and to decrease PPE-use. However, it was unknown whether using the continuous monitoring device indeed led to early detection of patient deterioration and could be clinically useful in the future to assess patients’ vital status over short time periods. Therefore, the aim of this study was to determine whether continuously monitoring heart rate and respiratory rate remotely would reliably detect deterioration earlier, or conversely, if using such a device mostly leads to false re-assurance or false alarms in COVID-19 patients. Additionally, interrater agreement was assessed.

## Methods

### Study Design

We conducted a cohort study in a large topclinical hospital in the Netherlands. We included all patients ≥ 18 years of age, who had been admitted to the hospital for COVID-19 infection between May 2020 and February 2021 and received a sensor during their stay in the hospital. A COVID-PCR-test, as well as blood and sputum cultures were performed upon hospital admission to confirm the diagnosis. Patients received a sensor, which continuously measures heart rate and respiratory rate, when available. All patients were asked for permission to use their data for research upon admission to the hospital, which was registered in the Electronic Medical Record (EMR). Permission to use the data for research was confirmed via phone calls after discharge. The study fell outside the remit of the law for Medical Research Involving Human Subjects Act and was approved by the local ethical committee (Reference number 2020 − 1733).

## Patient Care

All patients with COVID-19 were hospitalized when supplemental oxygen was required to maintain an SpO2 > 94% in a dedicated ward. Treatment consisted of daily 6 mg dexamethasone for 10 days, and daily 2850 IE nadroparine was administered for thromboprophylaxis. Oxygen was supplemented by nasal cannula, face mask or High Flow Nasal Cannula (HFNC) up to 60 L/min with a maximum Fraction of inspired oxygen of 60%, in increasing order.

Monitoring consisted of measurement of the level of consciousness, heart rate, respiratory rate, automated non-invasive blood pressure, body temperature, and oxygen saturation by a nurse every 4 h, resulting in a modified EWS (MEWS). A patch (Biosensor, Philips N.V., Eindhoven, The Netherlands) measured heart rate and respiratory rate continuously (every 4 s) and  was placed on the left anterior axillary line, at the 6th intercostal space, approximately. The nursing staff used these measurements for the MEWS. These signals were visible on a central monitoring post on the nursing station as well, but it was stressed that continuous vital sign monitoring was not the objective of the sensor. Alarms were disabled and the nursing station was not continuously operated.

An attending physician visited the patients at least daily and was available for questions from the nursing staff. For clinical deterioration, this physician was contacted first. If a medical emergency occurred, the nursing staff or the attending physician could contact a rapid response team (RRT) directly. This RRT, consisting of intensive care unit (ICU) nurses and physicians, was available 24/7, and the nursing staff from the ward was able to contact this team immediately, based on the MEWS-score [18]. Patients were admitted to the ICU when the attending intensivist deemed it necessary, at which time the sensor was removed. Typically, a fraction of inspired oxygen of high flow nasal cannula greater than 60% was a reason for ICU-admission.

## Sensor Events and EMR Events

**Fig. 1:**
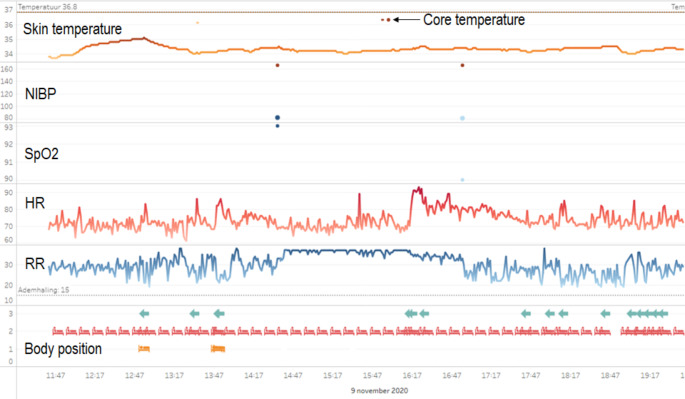
Screen capture of the dashboard used by the ICU physicians to assess the continuous measurements

Two intensive care physicians (RVP and MVK, Assessor 1 and Assessor 2) independently assessed the continuous HR and RR measurements. Continuous measurements of HR and RR were provided by the Biosensor. Identities of the patients were pseudonymized, and the physicians did not have access to the master file that linked patients’ research IDs to their identities. The HR and RR measurements were presented as cases of 8-hour periods. Thus, multiple cases were produced for each patient, which were assessed chronologically. The cases also included the latest known SpO_2_ and blood pressure levels at the start of the case, as well as age, height, and weight of the patient. Data were presented in a dashboard view, as shown in Fig. 1. In this dashboard, physicians could zoom in and out, and scroll back to earlier cases of the same patient.

For each case, the physicians completed a study form where they indicated the following: (1) the case ID, (2) whether sensor data suggested clinical deterioration, (3) the date and time at which the clinical deterioration should be acted upon, and (4) the causative vital sign(s) for suspicion of clinical deterioration. As a general rule, it was decided that action should be taken when a change in either HR or RR of around 20% was observed over the course of at least 20 min. Henceforth, the data reported in the study forms is referred to as alarms.

After the continuous measurements of the sensor data had been assessed by both physicians, another physician (VJG), blinded to the sensor data assessments, independently reviewed the Electronic Medical Record (EMR). He noted all events that were registered during the time each patient was admitted for COVID-19 and was equipped with a sensor. Specifically, the events that were noted included ICU admissions, pharmaceutical interventions, deterioration-driven diagnostics, and increases in respiratory assistance. These events are referred to as EMR events.

## Outcomes

Primary outcomes of the present study were the number of true alarms and the number of false alarms. Alarms perceived within 4 h prior to or after an EMR event were considered true alarms. Alarms that did not meet this criterion were considered false alarms. Secondary outcomes included: (1) the time difference between true sensor alarms and EMR events, (2) interrater agreement for the sensor events, (3) the number of non-detected EMR events, and (4) the severity of non-detected events.

## Statistical Analyses

For baseline characteristics, we used the mean and standard deviation (SD) for continuous variables following a normal distribution, and the median and interquartile range (lower (LB) and upper boundary (UB)) for variables with a skewed distribution. Categorical variables are presented as numbers and percentages. We matched sensor alarms and EMR events within a 4-hour window of one another and calculated the time difference.

We considered the physicians’ assessment of the sensor data to concern the same alarm when they were within 90 min of one another. To calculate the interrater agreement, we used the total number of sensor alarms as the nominator, and the number of agreed alarms as the numerator.

We matched events using the data.table package in R Statistical Software version X.

## Results

Between May 2020 and February 2021, 63 patients received a sensor, 41 of which were excluded for analysis because of a negative test for COVID-19. The 22 included patients were monitored for a total of 1481 h.  Table 1 presents an overview of baseline characteristics, including baseline laboratory test results.

During continuous monitoring, five patients were admitted to the ICU, 3 of which died. Overall, 5 patients out of this cohort died during hospital admission.

**Table 1:** Baseline characteristics of 22 patients with COVID-19

Age *years*	59.8	(16.6)
Female	8	(38%)
Pregnant	1	(5%)
Do Not Resuscitate	4	(19%)
BMI *kg/m*^*2*^	28	(27–32)
Length of stay *days*	11	(7–22)
Admission day (day of sickness)	8.4	(3.6)
Continuous monitoring duration (hours)	75	(34–93)
Admitted to ICU	6	(27%)
Death		
During admission	5	(23%)
During continuous monitoring	2	(10%)
Comorbidities		
Chronic Obstructive Pulmonary Disease	4	(19%)
Asthma	1	(5%)
Hypertension	8	(38%)
Diabetes	7	(33%)
Other	10	(48%)
None	6	(29%)
Oxygen therapy (L/min)	3	(1–5)
Laboratory test results upon hospital admission		
Hb *mmol/L*	8.1	(1.3)
WCC count * 10^*9*^*/L*	6	(4–10)
Urea *mmol/L*	74	(58–91)
Creatinine *µmol/L*	332	(273–413)
CRP *mg/L*	5	(4–9)
LDH *U/L*	95	(35–168)

*Data are reported as Mean (SD), n (%), or Median (LB IQR-UB IQR). Abbreviations: WCC = White blood Cell Count, CRP = C-Reactive Protein, LDH = Lactate Dehydrogenase*

## EMR Events

For 16 patients one or more events were noted in the EMR, for a total of 48 events. Ten (21%) of these events were detected by reviewing the continuous data (i.e. these were also noted as alarms). Table 2 shows an overview of the number of events per type, the number of unique patients in which those events occurred, and the events detected with the sensor data. The most severe events, which consist of ICU-admissions and RRT calls, were detected in 2 out of the 10 cases. Supplemental oxygen was increased 20 times, for 9 different patients, of which only 1 event was detected with the sensor data. Pharmaceutical interventions consisted of one case of morphine administration and one case of starting palliative care. Online Resource 1 details the actions comprising the combined events.

**Table 2:** Number of events registered in the electronic medical record (n = 48) per type of event, detection of EMR events with sensor data, and number of unique patients in whom events occurred

Event	Number of events	Detected with sensor data	Number of unique patients
ICU admission	6	0	6
RRT Calls	4	2	4
Combined event	12	4	6
Pharmaceutical intervention	2	1	2
Supplemental oxygen	20	1	9
Fever / sepsis	1	0	1
Diagnostics	1	0	1
Unplanned check-up	2	2	2
Total	48	10	16

## Sensor Alarms

The intensive care physicians indicated a total of 62 alarms based on sensor data, across 17 patients. Of the 62 alarms, 6 were scored by both physicians (interrater agreement 10%). Of the 17 patients with alarms, 13 also had one or more EMR events. Of the 62 alarms, 13 (21%) were also noted in the EMR, and thus were true alarms, while 49 (79%) were false alarms. Table 3 shows the 13 true alarms in more detail, with the type of EMR event, including the time difference between the alarms and the EMR events.

**Table 3:** Overview of 13 true sensor alarms, i.e. sensor alarmss that were within 4 h of EMR events

Event type EMR	EMR Description	Time difference Assessor 1 (minutes)*	Time difference Assessor 2 (minutes)*
Check-up	Check-up by physician related to fever and nausea	-90	-153
Combined event	Delirium, removes NR mask, increased dosage antipsychotics	-125	+ 80
Combined event	Check-up by physician, oxygen increased	-110	+ 236
Supplemental oxygen	Oxygen increased	+ 42	Not detected
RRT call	RRT call related to high demand for supplemental oxygen	+ 20	Not detected
Severe combined event	RRT call, start antibiotics, administered diuretics, start non-invasive ventilation	+ 17	Not detected
Serious medical event	Morfine administered	-70	Not detected
Check-up	Check-up by physician related to tachypnea, no change in policy	-95	Not detected
Combined event	Check-up by physician, administered diuretics and morfine	Not detected	-115
Combined event	Check-up by physician, oxygen increased	-144	Not detected

*A negative time difference means that events were detected earlier with sensor data than with conventional monitoring. A positive time difference means that events were detected later with sensor data than with conventional monitoring.

Of the 13 true alarms, 8 were detected earlier than the time registered in the EMR, while 5 were detected later. The mean time between sensor and EMR events (n = 13) was − 39 (SD = 113) minutes, meaning that on average the true alarms were perceived before action was taken in clinical practice.

A total of 10 sensor alarms were perceived in patients who were admitted to the ICU or for whom an RRT call was placed at any point during the monitoring period, compared with 52 in patients for whom RRT calls or ICU admissions were not necessary. Timelines showing all alarms and EMR events are provided in Online Resource 2, and timelines for all alarms and only severe EMR events are provided in Online Resource 3.

Of the 38 EMR events that were not detected with the sensor, 8 (21%) were ICU admissions or RRT calls. This is comparable to the 2 severe events out of the 10 EMR events that were detected (20%).

## Discussion

The present study shows that clinical deterioration was not reliably detected by continuously monitoring respiratory and heart rate in hospitalized patients with COVID-19. Approximately a fifth (10 out of 48) of the events reported in the EMR were detected by assessing the sensor data, and a similar proportion of severe events alone (2 out of 10). At the same time, the amount of false alarms (i.e. physicians noting deterioration by viewing the sensor data but no reporting of any event in the EMR) was high (79%). The interrater agreement was only 10%.

Continuous monitoring is emerging on the wards, with many monitoring solutions offering different measurements. Respiratory rate and heart rate are the most sensitive predictors of deterioration in many types of disease, but not for COVID-19. The low number of true alarms can be explained considering the pathophysiology of the disease, which was unknown in the beginning of the pandemic when we used the sensor device in this population. COVID-19 patients are often referred to as “happy hypoxics”, with a pure hypoxic respiratory insufficiency [13, 14]. Since the hypoxemia is treated with supplemental oxygen, the threshold of the hypoxemic ventilatory response is not reached, leading to an unaffected heart rate and respiratory rate. This may only occur when hypoxemia reaches the threshold or in case of insufficient tissue oxygenation. Since HR and RR remain unaffected for a long period of time, continuous monitoring of these vital signs is unlikely to lead to detection of clinical deterioration. As such, we hypothesize that continuous monitoring SpO2 would improve detection of clinical deterioration in COVID-19 patients. This illustrates that the choice of a monitoring solution should be based on the expected pathophysiology of the patient population, rather than the availability of devices.

Moreover, this study does not only demonstrate a lack of benefit of continuous monitoring in COVID-19 patients, it also demonstrates a harmful effect, with respect to additional workload and alarm fatigue. Based on two IC physicians’ clinical impression of the continuous data, 79% of the sensor-based notifications did not correlate with clinical deterioration. This would lead to additional check-ups, which may be unnecessary, consequently leading to alarm fatigue. Even though we assume that the EMR-based events were always correct, one may argue that the sensor-based events were correct, but missed in clinical practice. If so, this would improve the timeliness of check-ups and possibly more timely treatment. However, no clinically relevant changes were detected within a window of ± 4 h to a sensor-based notification. This supports the assumption that the EMR-based events were correct.

The low interrater agreement illustrates the difficulty of assessing COVID-19 patients’ health status based only on heart rate and respiratory rate, without a clinical view of the patient. It also implies that interpretation of the data and subsequent action are likely to vary from one physician to another. Even though the physicians were guided with the rule of thumb to use a 20% deviation from steady state, the absence of gold standards clearly affected their interpretation of the data. Furthermore, computer-based analysis or machine learning might eliminate the physicians’ interpretation, but this would require much more data and limits its use in a relatively new disease such as COVID-19. In predicting COVID-19 severity and ICU admission new scores including not only vital signs, but also patients’ demographic data, comorbidities, oxygen requirement and laboratory results, are being developed, but not yet leading to optimal results [19].

Our study questions the idea that patients’ condition can be comprehended fully using only continuous measurement of commonly measured vital signs. Literature shows that nurses and physicians alike frequently mention the importance of their clinical judgement in detecting deterioration of patients [20–22]. It is no surprise, then, that various EWSs include a range of aspects reflecting clinical judgement in their calculation [23]. The present study shows that continuously monitoring vital signs does not improve detection of clinical deterioration when used as a replacement for clinical judgement. As such, it seems that not all relevant information is captured with continuous measurement of vital signs. Elsewhere, a system that also incorporates visual monitoring of patients has been shown to reduce mortality and length of stay [24, 25]. Thus, using continuous measurement of vital signs in combination with visual monitoring may further improve detection rates of clinical deterioration. Therefore, we would advocate using continuous monitoring as an addition to clinical judgement, especially in hospitalized patients, in which deterioration occurs more often than in out-patients.

## Strengths and Limitations

While the strengths of the present study include blinded and independent assessment of the sensor data by two physicians (without knowledge of the EMR data) and the EMR extraction by a third, blinded physician (without knowledge of the sensor data), there are limitations to consider as well. First, the number of individual patients is low, even though it led to sufficient data and clinical events. Second, the low interrater agreement indicates that the interpretation of data by ICU-physicians is an uncontrolled potential of bias in the absence of guidelines on how continuous measurements should inform treatment decisions. Third, the method of sensor-data assessment does not reflect clinical practice. In clinical practice, the continuous data will probably be reviewed upon clinical indication, while in this study the data was assessed in 8-hour-timeframes. Still, the approach taken in this study does reflect the current clinical practice of using threshold values to infer clinical deterioration, without advanced computational techniques such as machine learning. Fourth, we defined a true alarm as being a sensor-based alarm within a window of 4 h of an EMR-based event. This was chosen because of the frequency of 6 nursing check-ups a day and our consideration that an event was > 4 h apart, it would become more likely that this was a non-corresponding event. Choosing a different window would change the numbers of true and false alarms. Fifth, as mentioned earlier, we used the EMR-data as the ground truth, but the EMR data may be unreliable as well. It is possible that not all events were detected or noted in the EMR, or that times noted were inaccurate. Still, any events that were not reported are likely to have been mild events. It is unlikely that significant events such as RRT calls or ICU admissions were not reported in the EMR. While we believe that these limitations affect the precision of this study, it does not affect the overall outcome. Continuously monitoring heart rate and respiratory rate is unreliable in COVID-19 patients with respect to detection of deterioration, when the data are assessed by ICU physicians.

Future research should investigate whether there are other conditions in which continuous monitoring of heart rate and respiratory rate alone is sufficient to detect deterioration. Additionally, whether monitoring of additional vital signs or the use of clinical information could lead to better detection rates in COVID-19 patients could be the focus of another study. For example, it is possible to use combinations of summary measures of vital sign data in a clinical prediction score for COVID-19 patients [26]. Finally, machine learning methods might provide improve detection of patient deterioration compared with assessment by ICU physicians [27, 28].

## Conclusion

The present study shows that continuously wireless monitoring heart rate and respiratory rate in COVID-19 patients does not reliably detect clinical deterioration when assessed by ICU physicians. In fact, it may lead to a low detection of clinical deterioration and a high number of false alarms. Monitoring solutions for selected conditions should be selected on pathophysiology of the condition.

## Electronic Supplementary Material

Below is the link to the electronic supplementary material.

Supplementary Material 1

Supplementary Material 2

Supplementary Material 3

## Data Availability

No supporting data is available.
