# Supplementary figures and images for: Detecting Patient Deterioration Early Using Continuous Heart rate and Respiratory rate Measurements in Hospitalized COVID-19 Patients
Source: J Med Syst. 2023 Jan 24;47(1):12. doi: 10.1007/s10916-022-01898-w (PMC9871416; doi:10.1007/s10916-022-01898-w)

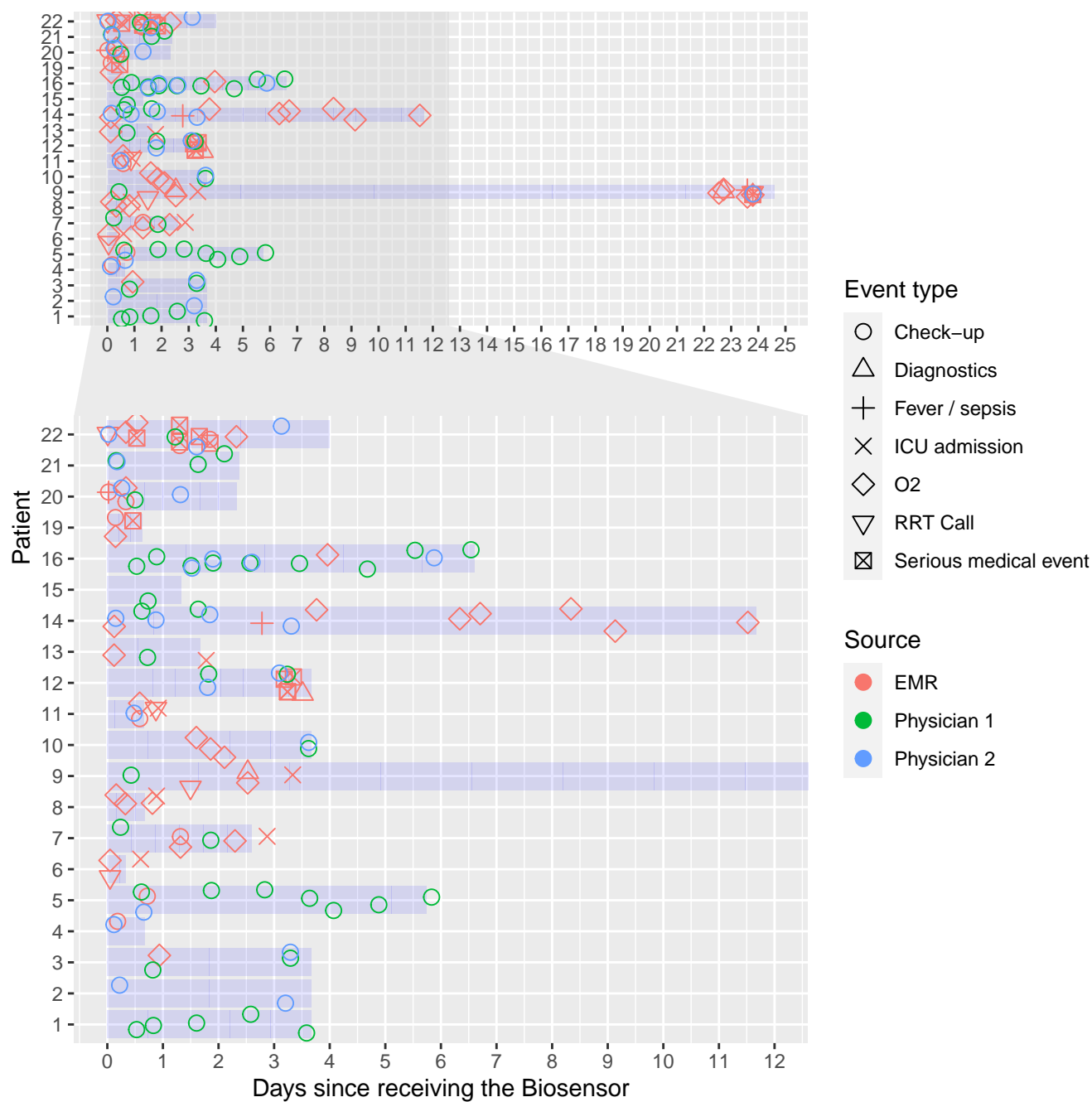

Supplement: Supplementary file 1 — Supplementary Material 1 [file 10916_2022_1898_MOESM1_ESM.pdf]

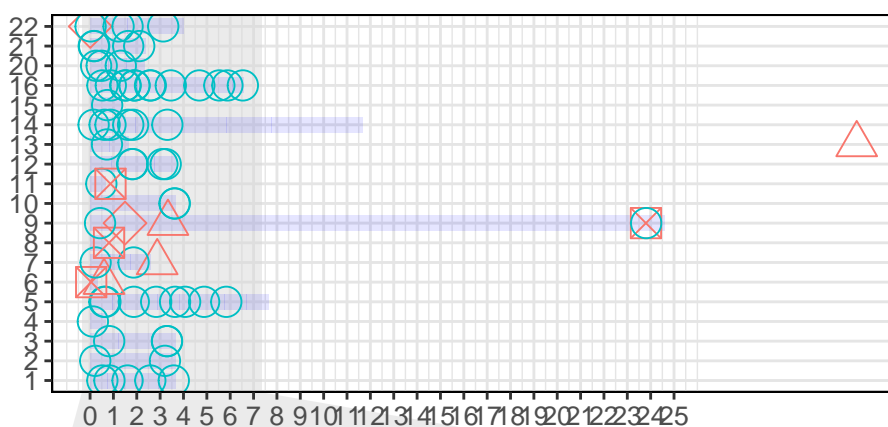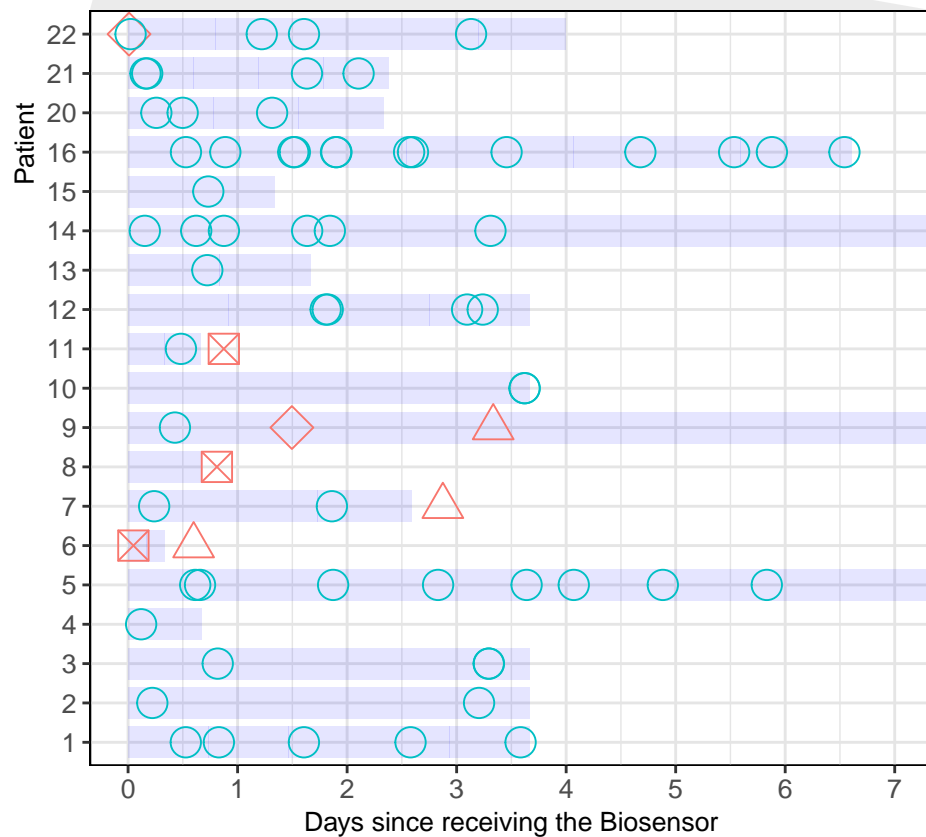

- 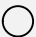 Check-up
- 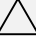 ICU admission
- 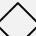 RRT Call
- 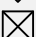 Severe combined event
- 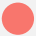 EMR
- 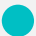 Physician

Supplement: Supplementary file 3 — Supplementary Material 3 [file 10916_2022_1898_MOESM3_ESM.pdf]
