# Supplementary material for: Detecting Patient Deterioration Early Using Continuous Heart rate and Respiratory rate Measurements in Hospitalized COVID-19 Patients
Source: J Med Syst. 2023 Jan 24;47(1):12. doi: 10.1007/s10916-022-01898-w (PMC9871416; doi:10.1007/s10916-022-01898-w)
Supplement: Supplementary file 2 — Supplementary Material 2 [file 10916_2022_1898_MOESM2_ESM.docx]

**Online Resource 1.** Clarification of the combined events

| Event | Action | Event type |
| --- | --- | --- |
| Combined event 1 | Delirium, removes NR mask | Serious medical event |
|  | Increased delirium | Serious medical event |
|  | Administered antipsychotics | Serious medical event |
|  | Increased delirium | Serious medical event |
| Combined event 2 | Unplanned check-up | Check-up |
|  | Increased supplemental oxygen | Oxygen |
| Combined event 3 | Unplanned check-up | Check-up |
|  | Blood culture | Fever / sepsis |
| Combined event 4 | Unplanned check-up | Check-up |
|  | Increased supplemental oxygen | Oxygen |
| Combined event 5 | Increased supplemental oxygen | Oxygen |
|  | Administered diuretics | Serious medical event |
| Combined event 6 | Unplanned check-up | Check-up |
|  | Administered diuretics | Serious medical event |
|  | Administered morphine | Serious medical event |
| Combined event 7 | Unplanned check-up | Check-up |
|  | Start palliative care | Serious medical event |
| Combined event 8 | Unplanned check-up | Check-up |
|  | Increased supplemental oxygen | Oxygen |
| Combined event 9 | X-ray thorax | Diagnostics |
|  | Increased supplemental oxygen | Oxygen |
| Combined event 10 | X-ray thorax | Diagnostics |
|  | Increased supplemental oxygen | Oxygen |
| Combined event 11 | Increased supplemental oxygen | Oxygen |
|  | Blood culture | Fever / sepsis |
| Combined event 12 | Unplanned check-up | Check-up |
|  | Increased supplemental oxygen | Oxygen |
| Severe combined event 1 | RRT call | RRT call |
|  | Start antibiotics | Fever / sepsis |
|  | Increased supplemental oxygen | Oxygen |
|  | Administered diuretics | Serious medical event |
| Severe combined event 2 | RRT call | RRT call |
|  | Increased supplemental oxygen | Oxygen |
| Severe combined event 3 | Increased supplemental oxygen | Oxygen |
|  | ICU admission | ICU admission |
| Severe combined event 4 | RRT call | RRT call |
|  | ICU admission | ICU admission |
